# Placental Nutrient Transport in Gestational Diabetic Pregnancies

**DOI:** 10.3389/fendo.2017.00306

**Published:** 2017-11-07

**Authors:** Marisol Castillo-Castrejon, Theresa L. Powell

**Affiliations:** ^1^Division of Reproductive Sciences, Department of Obstetrics and Gynecology, University of Colorado Anschutz Medical Campus, Aurora, CO, United States; ^2^Department of Pediatrics, Section of Neonatology, University of Colorado, Aurora, CO, United States

**Keywords:** obesity, gestational diabetes, placental transport, syncytiotrophoblast, fetal growth

## Abstract

Maternal obesity during pregnancy is rising and is associated with increased risk of developing gestational diabetes mellitus (GDM), defined as glucose intolerance first diagnosed in pregnancy (1). Fetal growth is determined by the maternal nutrient supply and placental nutrient transfer capacity. GDM-complicated pregnancies are more likely to be complicated by fetal overgrowth or excess adipose deposition *in utero*. Infants born from GDM mothers have an increased risk of developing cardiovascular and metabolic disorders later in life. Diverse factors, such as ethnicity, age, fetal sex, clinical treatment for glycemic control, gestational weight gain, and body mass index among others, represent a challenge for studying underlying mechanisms in GDM subjects. Determining the individual roles of glucose intolerance, obesity, and other factors on placental function and fetal growth remains a challenge. This review provides an overview of changes in placental macronutrient transport observed in human pregnancies complicated by GDM. Improved knowledge and understanding of the alterations in placenta function that lead to pathological fetal growth will allow for development of new therapeutic interventions and treatments to improve pregnancy outcomes and lifelong health for the mother and her children.

## Introduction

The global prevalence of obesity is increasing in both advanced and emerging countries. With a high prevalence among women of reproductive age, obesity-related complications during pregnancy such as gestational diabetes mellitus (GDM) are also increasing (2). Although obesity is considered a risk factor for developing GDM, deciphering the independent effects of maternal obesity and GDM are not fully understood. The placenta constitutes the maternal–fetal interface responsible for the transfer of nutrients, thereby contributing to fetal growth *in utero* (3). GDM is associated with an increased risk of fetal overgrowth, where placental nutrient transport may be altered. This review describes placental nutrient transport of glucose, amino acids, and lipids in human pregnancies complicated with GDM.

## The Obesity Epidemic

The obesity epidemic represents a major public health concern with a negative impact on morbidity, mortality, quality of life, and results in significant economic cost in treatment and prevention (2). In 2014, the World Health Organization (WHO) reported that more than 1.9 billion adults (39%) were overweight and over 600 million (13%) are obese (4). Worldwide prevalence of obesity in women is higher than in men, at 15 and 11% respectively. In the U.S., the 2013–2014 National Health and Nutrition Examination Survey (NHANES) estimated that 35.0% of men and 40.4% of women are obese. Obesity is a multifactorial disease with behavioral, genetic, socioeconomic, and environmental origins that predisposes individuals to numerous adverse metabolic disorders, including cardiovascular diseases, type 2 diabetes, musculoskeletal disorders, infertility, liver diseases, psychological conditions, sleep disorders, and some types of cancer (5, 6). The prevalence of obesity in women of childbearing age (18–44 years old) is 27.2% (7). When almost one-third of childbearing-age women enter pregnancy obese, there is a critical need to examine the effects of obesity on short- and long-term maternal and offspring health.

Maternal pre-pregnancy obesity has been associated with maternal and neonatal complications, such as GDM, hypertensive disorders, preeclampsia, medically induced preterm birth, macrosomia, large for gestational age (LGA), fetal defects and congenital anomalies, surgical complications, longer maternal length of hospital stay, maternal hemorrhage, and infection (8–11). Children born to obese mothers or women with excessive gestational weight gain have a higher risk of obesity, type 2 diabetes, and cardiovascular disease (12). Although childhood obesity is multifactorial and not fully understood, it is considered a risk factor for obesity in adolescence and adult life. Since the obese mother–infant dyad who develop metabolic complications during pregnancy, such as GDM, have higher risk of developing cardiovascular and metabolic disorders later in life (13), this in turn leads to transgenerational transmission of disease.

## Gestational Diabetes Mellitus

Pregnancy is a state characterized by profound metabolic and physiological changes to support fetal development and growth (14). As an adaptation in healthy pregnancy, maternal insulin sensitivity and insulin-mediated glucose consumption in peripheral tissues, including skeletal muscle and adipose tissue declines (15). The placenta secretes hormones such as estrogens, progesterone, growth hormone, and human placental lactogen which may contribute to the establishment and maintenance of pregnancy by regulating the maternal physiological adaptations to pregnancy (16). In pregnancy, insulin secretion increases due to hyperplasia of beta cells in the pancreas (17). Despite the increase in insulin secretion, pregnancy is characterized by a relative insulin resistance state, particularly in the third trimester, which favors the metabolic needs of the developing fetus (18). Pregnancies complicated by GDM are characterized by glucose intolerance first recognized in pregnancy, where maternal pancreatic β-cells are not able to secrete sufficient insulin to maintain normal glycemia in the mother (19).

There is no international agreement about the definition of GDM, and several diagnostic criteria are used worldwide (1). The American Diabetic Association defines GDM as a glucose intolerance diagnosed in the second or third trimester of pregnancy not related to type 1 or type 2 diabetes mellitus (20). The National Institute for Health and Care Excellence defines GDM as a fasting plasma glucose ≥5.6 mmol/L or a 2-h plasma glucose level ≥7.8 mmol/L. Although the prevalence of GDM varies among countries, the global prevalence is estimated to be 14% of all pregnancies and is increasing along with the obesity epidemic (21).

**Figure 1:**
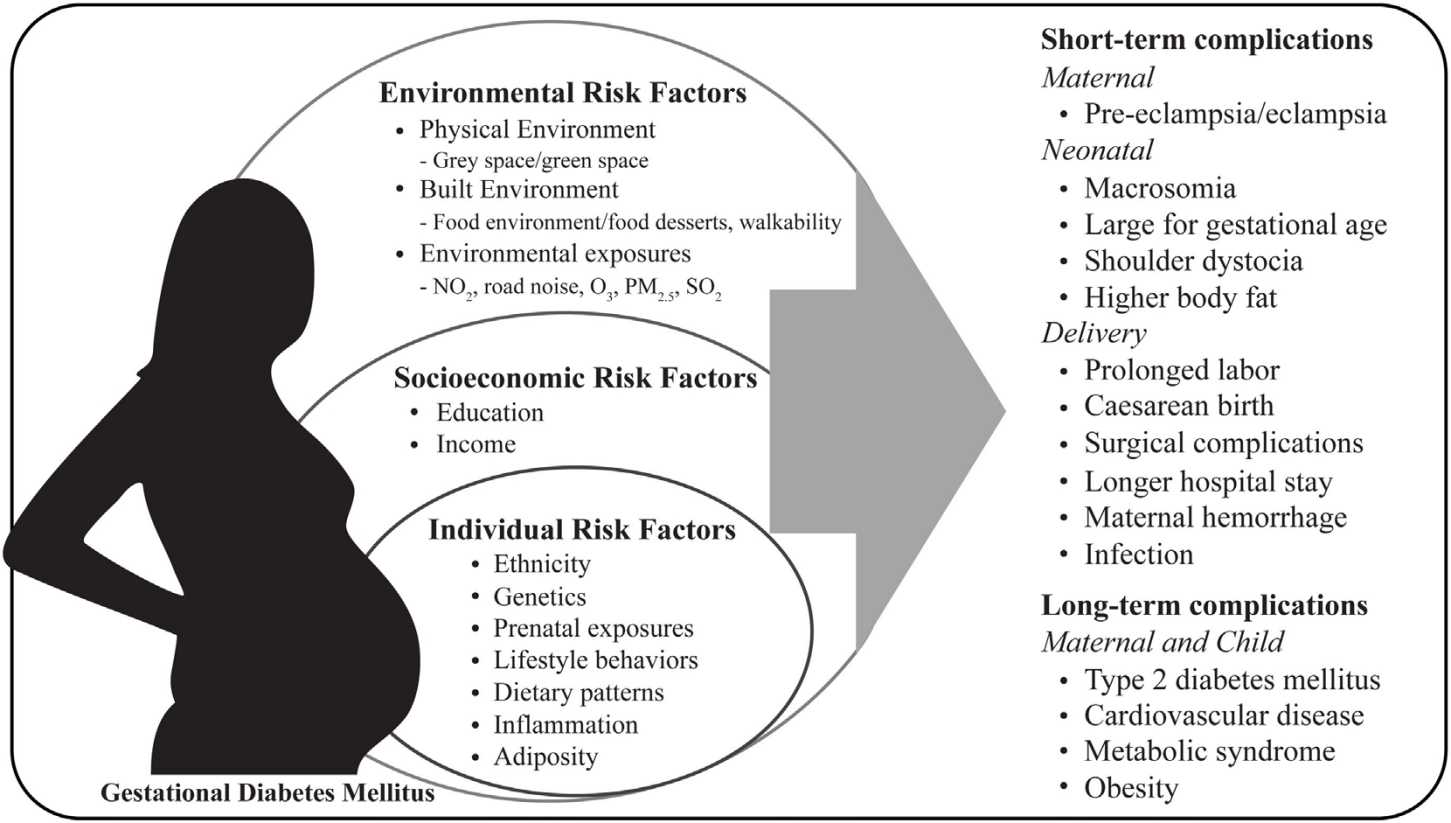
Maternal risk factors and pregnancy complications associated with gestational diabetes mellitus. NO_2_, nitrogen dioxide; O_3_, ozone; PM_2.5_: particulate matter; SO_2_, sulfur dioxide.

The increase in the prevalence of diabetes, including GDM, coincides with an increase in urbanization, reduced size and access to green spaces leading to a reduction in physical activity, excess gestational weight gain, changes in dietary patterns, exposure to environmental contaminants (e.g., nitrogen dioxide, ozone, particulate matter, and sulfur dioxide) and obesogenic environments (e.g., food environments and food desserts) (22–26). Pregnancies affected by GDM show increased risk of short and long-term complications for both mother and child (Figure 1). In the short term, GDM is associated with maternal hypertensive disorders during pregnancy, cesarean section delivery and lower rates of breastfeeding (19, 27, 28). It is well established that women diagnosed with gestational diabetes are at greater risk of developing GDM in future pregnancies, furthermore these women have long-term consequences such as type 2 diabetes, cardiovascular disease, and metabolic syndrome (29, 30). Infants born to GDM mothers compared to infants born from uncomplicated pregnancies, neonates are at increased risk for macrosomia, neonatal hypoglycemia, hyperbilirubinemia, shoulder dystocia, and have higher percentage of body fat (31). In the long term, offspring are also prone to developing obesity, type 2 diabetes, and other chronic metabolic disorders later in life (32–34). However, some studies have shown opposing findings where fetal birth weight was inversely correlated with type 2 diabetes risk dependent on ethnicity (35). Fetal overgrowth is likely due to increased nutrient delivery to the fetus and maternal hyperglycemia, hyperleptinemia, hyperinsulinemia, dyslipidemia, reduced adiponectin, and pro-inflammatory cytokines which can induce functional and structural abnormalities in the placenta and have been shown to modify the transport of macronutrients to the fetus (36–44).

## Placental Nutrient Transport in GDM

The placenta is a transient multifunctional organ responsible for nutrient transport from the mother to the fetus. The diabetic environment of a GDM pregnancy alters the development of the placenta and its function which have detrimental consequences on fetal development and growth (45). The effect of GDM on placental anatomy is not fully understood; however, morphological alterations have been described, such as maternal vascular malperfusion, fetal thrombosis, an imbalance of vasoactive signaling molecules, and enhanced oxidative stress (46–49).

In general, placentas from GDM mothers are frequently larger, however, placental shape, area, umbilical cord insertion, and number of terminal villi are not different when compared to placentas from healthy pregnancies. Syncytiotrophoblast surface has been shown to be increased and the villi are hypervascularized resulting in a larger feto-placental endothelial surface in diabetic placentas (50). Increased placental weight and placental-weight to birth-weight ratio have been described in pregnancies complicated with GDM with optimal maternal glycemic control (51). Placental abnormalities may be related to maternal glycemic control but further research is required to determine timing and causes of placental anatomical changes.

One report demonstrated that GDM is associated with increased placental endoplasmic reticulum (ER) stress. Although not specifically studied in the placenta, there are a number of links between ER stress and nutrient transport (52). For example, ER stress typically results in the phosphorylation of the translation initiation factor 2 eiF2α leading to inhibition of global protein translation and mTOR signaling, which is expected to inhibit nutrient transport. At the same time, however, eiF2α phosphorylation also increases ATF4 transcription, which in some cells promotes the transcription of specific amino acid transporters. Thus, ER stress in the GM placenta may contribute to changes in nutrient transport (53, 54).

The placenta is a fetal organ; therefore, the predisposition and frequency in metabolic disease such as obesity and type 2 diabetes may be mediated by sex-specific placental adaptations (55). Epigenetic and transcriptomic analysis demonstrates that placental transporter gene expression differs in a sex-dependent manner in response to maternal diet, metabolic disease, and across gestation (56–59). Although, sex differences in fetal growth are likely mediated by sex-specific placental function, studies evaluating placental nutrient transport changes by gender in pregnancies complicated by GDM have not been reported in the literature.

Gestational diabetes mellitus is associated with an altered maternal environment, which includes changes in circulating adipokines, growth hormones, and insulin, all of which can influence placental development and function. Placental functional adaptations, including abundance, localization, and functional modification of macronutrient transporters, occur in response to the metabolic intrauterine environment in GDM (60). Placental nutrient transporters are localized to the syncytiotrophoblast, the multinucleated epithelial barrier comprised of the microvillous plasma membrane (MVM) facing the maternal circulation and basal plasma membrane (BM) directed toward the fetal circulation. Alterations in macronutrient transfer in the placenta of GDM pregnancies is reviewed below and summarized in Table 1.

**Table 1:** Changes in the expression level (protein or mRNA) of placental nutrient transporters in the human placenta from pregnancies complicated with gestational diabetes mellitus.

Nutrient	Transporter	SLC	Substrate	Group	Tissue	Localization	Protein expression	Reference
Glucose	GLUT1	*SCL2A1*	Glucose Galactose Glucosamine	GDMG1	Syn	MVM	↔	(61, 62)
						BM	↔, ↑	(61, 62)
				GDMG2	Syn	MVM	↔, ↑	(61, 62)
						BM	↔, ↑	(60–63)
				Glyburide	Syn	MVM	↑	(64)
						BM		

	GLUT4	*SCL2A4*	Glucose	GDMG1	Syn		↔	(60, 63)
			Glucosamine	GDMG2			↓, ↑	(60, 63)

	GLUT9			GDMG1	Syn		↑	(65)
				GDMG2			↑	
	GLUT9a	*SCL2A9*	Glucose	GDMG1,		MVM	↔	
			Fructose	GDMG2		BM	↑	
	GLUT9b		Urea	GDMG1,		MVM	↑	
				GDMG2		BM	↔	

Neutral amino acids	System A				Syn	MVM	↑, ↔*	(66, 67)
	SNAT 1	*SLC38A1*	Alanine					
	SNAT2	*SLC38A2*	Serine					
	SNAT4	*SCL38A4*	Glutamine			BM	↔*	

	System L				Syn	MVM	↑, ↔	
	LAT1	*SLC7A5*	Leucine					
	LAT2	*SLC7A8*	Phenylalanine			BM	↔	

Lipids	LPL	*LPL*			Syn	MVM	↑, ↔	(68)
					PH		↓	(69)
	EL	*LIPG*			PH		↑	(70)
	FATP	*SLC27*						
	FATP1	*SLC27A1*	Free Fatty Acids		PH		↓	(68)
	FATP4	*SLC27A4*	Long-chain fatty acids					
	FATP6	*SLC27A6*					↑	
	FABP				Syn	Cytoplasm	↑	(71, 72)
	L-FABP	*FABP*						
	FABP4							
	FABP5							

BM, Basal membrane; MVM, microvillous membrane; PH, placenta homogenate; Syn, syncytiotrophoblast; GDMG1, diet-controlled gestational diabetes mellitus; GDMG2, insulin-controlled gestational diabetes mellitus; *, transport activity; ↑, increased; ↓, decreased; ↔, unaltered.

### Glucose Transport

Glucose is a primary energy source for the fetus and must be obtained from the maternal circulation due to low fetal gluconeogenesis *in utero*. To fulfill fetal demands maternal–fetal transport of glucose takes place by facilitated diffusion through sodium-independent glucose transporters (GLUTs), a specialized type of transmembrane protein. Glucose transport in the placenta is regulated by the extracellular (maternal) glucose concentration and GLUT expression and activity in the MVM (glucose uptake) and BM (glucose delivery). Net glucose delivery is primarily dependent on the concentration gradient from mother to fetus, placental cellular metabolism and GLUT1 (*SLC2A1*) expression, particularly in the BM. Several GLUT isoforms have been described in placenta: GLUT1 (*SLC2A1*), GLUT3 (*SLC2A3*), GLUT4 (*SLC2A4*), GLUT8 (*SLC2A8*), GLUT9 (*SLC2A9*), and GLUT12 (*SLC2A12*) with variation that is gestational age dependent (60, 73). Transplacental glucose transfer can also be influenced by glucose metabolism of the placenta, such as gluconeogenesis, glycogenesis, and glycolysis, and utero-placental blood flow (74).

Insulin is essential for regulating intracellular and plasma levels of glucose in peripheral tissues, such as adipose tissue, skeletal muscle, and liver, *via* mechanisms that include Akt/PKB and mitogen-activated kinase (MAPK) pathways. The activation of the insulin signaling pathway in the placenta is associated with control of cell survival, differentiation, proliferation, and metabolism of primarily amino acids, and, in a gestational age-specific manner, to the transfer of glucose (75). In the first trimester of pregnancy, placental glucose transfer is enhanced by insulin; while during the third trimester glucose uptake is not regulated by insulin (76). As in maternal peripheral tissues, where insulin induces glucose uptake by triggering the translocation of the GLUT isoform GLUT4 to the plasma membrane, during first trimester of pregnancy GLUT4 is expressed but is downregulated in term placentas (77). At term glucose transfer from mother to fetus by the placenta is primarily through GLUT isoform GLUT1, present in very high abundance in the syncytiotrophoblast MVM to facilitate rapid uptake of glucose from the maternal circulation. The basal membrane has been identified as the rate-limiting step in placental glucose transport due to reduced expression of the transporter and lower surface area of the basal plasma membrane (78–80). GLUT1 expression in the BM increases twofold in late second trimester and then remains unaltered until term (81).

Pedersen’s original hypothesis proposed that maternal hyperglycemia in type 1 diabetes mellitus accelerates placental glucose transfer resulting in fetal hyperglycemia and hyperinsulinemia, which in turn stimulates fetal growth. However, macrosomia is also common in well-controlled diabetic pregnancies suggesting a change in placental function. Women with type 1 diabetes, with first trimester moderate hyperglycemia, showed higher expression of GLUT1 in the BM compared to healthy pregnancies (62). Likewise, a positive correlation has been reported between birth weight and GLUT1 density in the placental BM in type 1 diabetic pregnancies (82). Under *in vitro* conditions, hyperglycemia partially limits GLUT1 expression and its activity was inversely related to extracellular glucose in primary cultured human trophoblast from uncomplicated pregnancies (83). In addition, elevated glucose concentration promotes the translocation of the GLUT transporters from the cell surface to the intracellular compartment as a mechanism to downregulate glucose uptake (84) in cultured trophoblast cells. An interaction of insulin and glucose may be important in determining *in vivo* expression of placental GLUT isoforms.

Hyperglycemia has been proposed to be a major contributing factor to accelerated fetal growth. However, birth weight is not clearly correlated to maternal glycemic control among women with GDM or T1DM. Alterations in placental glucose transport capacity may explain the weak correlation. Conflicting results exist for GLUT1 expression and activity in MVM and BM of GDM with reports of no change (61) or twofold higher expression in the BM of GDM controlled with diet or diet plus insulin and, in a third study, d-glucose uptake was higher in the BM in all diabetic groups tested (62). Inconsistency in the data reported may be due to different methodologies and membrane fractions used, differences in criteria for GDM diagnosis, gestational age at diagnosis, maternal glycemic control, the effect of obesity and gestational weight gain, variation in treatment protocols, and small sample sizes. The limited and variable data on placental glucose transport in GDM-complicated pregnancies demonstrate the complexity of the disease process and the many regulatory factors that may impact placental function and fetal growth.

### Amino Acid Transport

Amino acid transfer across cellular membranes is mediated through either active transport processes, accumulative transporters, or exchangers. Amino acid concentrations are higher in the fetal than in maternal compartment, which reflects an active transport mechanism across the placenta. This is accomplished through secondary active transport *via* accumulative transporters that utilize energy provided by ion gradients such as sodium, chloride, and protons. Exchangers modify amino acid concentration by exchanging between intracellular and extracellular compartments (85). Most amino acids are transported between maternal and fetal circulation in both directions, while some are transported unidirectionally. For example, glutamate is transported from fetal liver to the placenta, where it is converted to glutamine and released back to fetal circulation to be utilized as a source of energy, anabolism, and nucleic acid synthesis (86).

Amino acid transport in the placenta is mediated by proteins expressed on the syncytiotrophoblast MVM and BM. The human placenta expresses more than 15 amino acid transport systems, where 7 are dedicated to neutral amino acids. Some amino acids are specifically transported by a single system, whereas others can be transported by multiple systems. System A consists of highly homologous subtypes of neutral amino acid transport proteins (SNATs) that facilitate the uptake of small non-essential neutral amino acids such as alanine, glycine, and serine. This system transports amino acids against their concentration gradient through co-transport with sodium into the cell. In human term placenta SNAT1 (*SLC38A1*), SNAT2 (*SLC38A2*), and SNAT4 (*SLC38A4*) are polarized to the MVM. Studies have shown that SNAT4 (*SLC38A4*) activity is higher during first trimester of pregnancy, while SNAT1 activity is upregulated at term (87). A recent study showed that SNAT1 is a major contributor to aminoisobutyric acid uptake, which is exclusively transported by system A, in human MVM-isolated vesicles (88). System A activity provides an abundant supply of small neutral amino acids to be exchanged for essential large neutral amino acids through another amino acid transporter, system L.

System L is a sodium-independent exchanger of branch-chain (such as l-leucine) and aromatic (such as l-phenylalanine) essential amino acids. It is a heterodimer constituted by a light chain, L-type Amino Acid Transporter, either LAT1 (*SLC7A5*) or LAT2 (*SLC7A8*) covalently attached to a heavy chain CD98/4F2hc (*SLC3A2*) (89). Non-essential amino acids are exchanged for essential amino acids enabling transport against their concentration gradient. LAT1 and LAT2 are both expressed in trophoblast cells from human term placenta and are present in the MVM with LAT2 also expressed in the BM (90).

Increased placental nutrient transport capacity in diabetes is associated with fetal overgrowth, however, the activity of amino acid transporters in placentas from diabetic women has not been well established and the available data are conflicting. System A activity, measured by aminoisobutyric acid uptake, has been described as unchanged in MVM isolated from placentas complicated by diabetes with normal fetal growth (91) and in MVM from placentas of macrosomic or appropriately grown newborns of diabetic women (67). By contrast, another study found system A and L activity in MVM was increased in pregnancies complicated with GDM and delivering LGA babies (66).

Importantly, placental transport of amino acids in GDM remains scarcely studied. Amino acid concentrations were increased in cord blood of well-controlled GDM pregnancies with normal birth weight while maternal amino acid concentration were not altered (92), suggesting that placental amino acid transport was increased in GDM pregnancies. Maternal insulin has no effect on the glucose transport in term placenta, however, activation of the insulin receptor in the placenta leads to activation of the nutrient sensoring system, mammalian target of rapamycin (mTOR). mTOR is a positive regulator of placental amino acid transport and stimulates cell proliferation and growth. Activation of mTORC1 signaling is associated with obesity and likely partially explains the increased size of placentas and fetal overgrowth, in both obese and GDM pregnancies (93). GDM is often complicated by obesity and current findings suggest an increase of placental capacity to supply amino acids in pregnancies complicated by maternal obesity and diabetes in particular when the fetus demonstrates accelerated growth (66, 94). Distinguishing the conditions that lead to fetal overgrowth in some but not all pregnancies complicated by metabolic disease remains elusive. Likewise, independent effects of obesity and GDM on placental amino acid transport function are unclear.

### Lipid Transport

Gestational diabetes mellitus is associated with maternal dyslipidemia characterized by high plasma triglycerides (TG) concentrations, low concentration of high-density lipoprotein cholesterol, and increased low-density lipoprotein cholesterol (69, 95). Thus, the placenta of pregnant women with obesity, and/or GDM is likely exposed to an excess supply of lipids that may contribute to greater transplacental transport, lipid storage, and/or lipotoxicity. In well-controlled GDM pregnancies, maternal lipids are strong predictors for higher fetal lipids and fetal growth (95). Larger newborns of GDM mothers have demonstrated higher free fatty acids (FFA), high insulin-to-glucose ratios and low TG levels compared to small or appropriate for gestational age infants of diabetic mothers. The same study showed that GDM is also associated with an increased fat mass in the neonate, which may be associated with altered placental function (95).

Fetuses require essential linoleic acid (18:2 n-6), α-linolenic acid (18:3 n-3) and long-chain polyunsaturated fatty acids (LC-PUFA) which cannot be synthesized by the placenta or fetus in adequate amounts to sustain normal growth. Therefore, maternal supply and placental transfer are crucial. Maternal lipoproteins are a main source of fatty acids (FA), either as non-esterified fatty acids (NEFAs) or esterified FA in TG and phospholipids for fetal supply. Placental FA transport requires the activity of TG hydrolases in the MVM of the syncytiotrophoblast. Lipases such as hormone sensitive lipase (HSL; *LIPE*), lipoprotein lipase (LPL; *LPL*), and endothelial lipase (EL; *LIPG*) generate NEFAs from maternal TGs. LPL and EL are the main lipases found and actively expressed in the placenta; with LPL localized in the MVM and EL also present in the membrane of capillary endothelial cells. NEFAs can cross the MVM by simple diffusion or facilitated by FA carriers such as FA translocase (FAT/CD36) and plasma membrane FA transport proteins (FATP1–6; *SLC27A1–6*). The FATP family consists of six related members of which five are expressed in human placenta (FATP1–4; *SLC27A1–4*) and FATP6 (*SLC27A6*) (96). Once in the cytoplasm FA are bound by cytosolic FA binding proteins (FABP; *FABP*) (97) which regulate the fate of FAs to either esterification, oxidation, or transfer to the fetus. Human trophoblast cells express FABP1, FABP3, FABP4, and FABP5 (98).

In pregnancies complicated by diabetes or GDM, placental transfer of lipids may be increased due to an increase of maternal–fetal concentration gradient of FFA and TG. This may explain fetal macrosomia and the increase of body fat mass in the newborns of diabetic women (99). LPL activity in MVM from diabetic pregnancies was found to be increased as well as the expression of liver FABP (L-FABP) in pre-gestation diabetic and GDM placentas (72). Furthermore, FABP4 and FABP5 expression was found to be increased in placentas from obese diabetic women when compared with placentas from obese, non-diabetic, or normal-weight women (71). Similarly, EL expression was upregulated in homogenates of placentas from obese-GDM pregnancies (70). In addition to the total amount of FFA required for normal fetal growth, the composition of lipids available to the fetus must be considered for appropriate fetal development. The proportion of polyunsaturated FA is reduced in arterial cord blood compared to venous in GDM fetuses, indicating enhanced utilization by fetal tissues rather than impaired placental transfer (100).

While evidence exists suggesting an increased placental capacity to deliver FAs to the fetus in the GDM pregnancy, work from Larque’s group suggests that the profile of FAs transported may be altered in these pregnancies. Using stable isotope labeled FAs orally administered 12 h before operative delivery, pregnancies complicated by GDM were found to have significantly lower n-3, 22:6 docosahexaenoic acid (80) in cord blood (101). In an additional study of placental transporter expression, they described lysophospholipid transporter MFSD2a, a potential DHA transporter, was reduced in the placenta of GDM women and MFSD2a expression correlated with cord levels of DHA (102). The regulation of lipid transporter expression in the placenta of GDM mothers is not known. These data suggest that the placenta may modulate FA delivery based on chain length and desaturation and in cases of GDM, the critically needed LC-PUFAs are not adequately transferred. The long-term consequences of this deficit are currently not known.

## Conclusion

Gestational diabetes and maternal obesity are both associated with increased risk of fetal overgrowth and accumulation of excess fat depots that contribute to lifelong health risk. This review points out that the role of the placenta in mediating these effects has not been adequately studied and existing data are conflicting. Consistent findings suggest that glucose transport capacity may be increased in GDM placentas and considering the potential for maternal hyperglycemia and greater post-prandial glucose excursions, the developing fetus is likely to be receiving excess glucose. Glucose entering the fetal compartment initiates pancreatic insulin release which functions to accelerate fetal growth and excess glucose is likely stored as fat. Amino acid transport is more complex and not well studied but recent evidence indicates that insulin signaling activates mTOR leading to greater amino acid transport capacity, once again supporting accelerated fetal growth. Maternal hyperlipidemia and alterations in placental FA transport protein expression suggest a potentially greater overall fetal FA delivery. Importantly, a deficit in DHA transfer, one of the LC-PUFA critically important for fetal brain development, has been described in GDM women. More research is needed to understand the role of the placenta in determining fetal growth trajectory in pregnancies complicated by both GDM and obesity and the independent effects of these metabolic disorders remain unclear. Early indications of which pregnancies complicated by metabolic disease will deliver large babies are critically needed. An increased mechanistic understanding of altered placental macronutrient transport function in GDM may lead to novel therapeutic interventions to modulate fetal growth and reduce lifelong health consequences.

## Author Contributions

MC-C and TP wrote and edited the manuscript.

## Conflict of Interest Statement

The authors declare that the research was concluded in the absence of any commercial or financial relationships that could be constructed as a potential conflict of interest. The reviewer AS-P declared a past co-authorship and shares the affiliation with the handling editor SO.
