# Corrigendum: Placental Nutrient Transport in Gestational Diabetic Pregnancies

**DOI:** 10.3389/fendo.2019.00005

**Published:** 2019-01-28

**Authors:** Marisol Castillo-Castrejon, Theresa L. Powell

**Affiliations:** ^1^Division of Reproductive Sciences, Department of Obstetrics and Gynecology, University of Colorado Anschutz Medical Campus, Aurora, CO, United States; ^2^Department of Pediatrics, Section of Neonatology, University of Colorado, Aurora, CO, United States

**Keywords:** obesity, gestational diabetes, placental transport, syncytiotrophoblast, fetal growth

In the original article, there was an error. The term “placental MVM” was used instead of “placental BM.”

A correction has been made to the **Introduction**, **Glucose Transport**, Paragraph Number three:

“Pedersen's original hypothesis proposed that maternal hyperglycemia in type 1 diabetes mellitus accelerates placental glucose transfer resulting in fetal hyperglycemia and hyperinsulinemia, which in turn stimulates fetal growth. However, macrosomia is also common in well-controlled diabetic pregnancies suggesting a change in placental function. Women with type 1 diabetes, with first trimester moderate hyperglycemia, showed higher expression of GLUT1 in the BM compared to healthy pregnancies (62). Likewise, a positive correlation has been reported between birth weight and GLUT1 density in the placental BM in type 1 diabetic pregnancies (82). Under *in vitro* conditions, hyperglycemia partially limits GLUT1 expression and its activity was inversely related to extracellular glucose in primary cultured human trophoblast from uncomplicated pregnancies (83). In addition, elevated glucose concentration promotes the translocation of the GLUT transporters from the cell surface to the intracellular compartment as a mechanism to downregulate glucose uptake (84) in cultured trophoblast cells. An interaction of insulin and glucose may be important in determining *in vivo* expression of placental GLUT isoforms.”

The authors apologize for this error and state that this does not change the scientific conclusions of the article in any way. The original article has been updated.

